# Light-dependant intraretinal ion regulation by melanopsin in young awake and free moving mice evaluated with manganese-enhanced MRI

**Published:** 2010-08-30

**Authors:** Bruce A. Berkowitz, Robin Roberts, David Bissig

**Affiliations:** 1 Department of Anatomy and Cell Biology, Wayne State University, Detroit, MI; 2 Department of Ophthalmology, Wayne State University, Detroit, MI

## Abstract

**Purpose:**

To test the hypothesis that in young, functionally blind mice, light-dependent intraretinal ion regulation occurs via melanopsin.

**Methods:**

Postnatal day (P) 7 wild type (WT, C57Bl/6) and melanopsin knockout (KO, opn4−/−, B6129) mice were light or dark adapted. Awake and freely moving animals were injected intraperitoneally (ip) with MnCl_2_. Four hours later, the mice in both groups were anesthetized and studied with manganese-enhanced MRI (MEMRI) to measure the extent of intraretinal uptake of manganese and whole retinal thicknesses.

**Results:**

In control P7 mice, light exposure increased (p<0.05) retinal manganese uptake over that in dark. This difference was observed throughout most of the retina. In P7 KO mice, intraretinal manganese uptake did not differ from that in age-matched dark-adapted WT mice, and was not light-dependent. No differences in whole retinal thickness were noted between groups.

**Conclusions:**

First time evidence is presented which demonstrates intraretinal ion regulation by melanopsin in vivo.

## Introduction

In the first ten days after birth, mice are functionally blind because their eyelids are fused, they have a prominent tunica vasculosa lentis and hyaloid circulations, and they lack synaptic connections between photoreceptors and ganglion cells. Nonetheless, changes in light levels can be detected in these young mice via functional intrinsically photosensitive retinal ganglion cells (ipRGC) [1,2]. These ipRGCs express the photopigment melanopsin, which depolarizes the cell membrane in response to light [3]. Direct investigation of retinal melanopsin physiology has largely been limited to ex vivo studies of isolated preparations and/or genetic manipulation.

These approaches have firmly established melanopsin as an important photopigment, but do not allow for analytical measurement of melanopsin-mediated activity in the retina of awake and freely moving animals.

At present, the only method available for measuring regional retinal ion regulation in vivo is manganese-enhanced MRI (MEMRI) [4-8]. Intraretinal uptake of manganese ion (Mn^2+^, a strong MRI contrast agent and biomarker for regulation of cations such as calcium), after systemic injection of a modest and nontoxic amount of MnCl_2_, is a surrogate biomarker of normal and diseased retinal function [4-8]. To date, the possibility of using MEMRI to investigate light-dependant melanopsin-induced activity has not been examined. In this study, we test the hypothesis that retinal uptake of manganese in young, functionally blind rodents is regulated by melanopsin.

## Methods

All animal experiments and procedures were approved by the Institutional Animal Care and Use Committee at Wayne State University and were in accordance with the NIH Guide for the Care and Use of Laboratory Animals. In all cases, mice were housed and maintained in a normal 12 h:12 h light-dark cycle.

### Animal groups

The following groups were examined with MEMRI at P7: C57BL/6 mice (WT, light adapted [n=5], dark adapted [n=7]), and melanopsin knockout (opn4^−/−^) B6129 mice (KO, light adapted [n=5], dark adapted [n=5]—a generous gift of Dr. David Berson). Light adaptation took place in normal laboratory lighting (about 300 lx).

### High-resolution MRI experiments

Before the MEMRI measurement, a manganese solution was injected intraperitoneally (ip; 66 mg MnCl_2_·4H_2_O/kg bodyweight) on the right side of each awake mouse, as previously described [8]. This manganese entered the circulatory system, crossed the blood retinal barrier, and entered retinal cells through, for example, L-type voltage gated calcium channels, in an activity-dependant manner [8,9].

Roughly 4 h after injection, plasma manganese content reached near pre-injection levels, and intraretinal manganese content was reasonably stable, and will be cleared slowly over several days [10]. Animals were maintained in either dark or continuous light for 4 h.

An MEMRI examination was then performed in darkness or dim red light using a 4.7 T Bruker Avance system to assess intraretinal manganese uptake. Immediately before each MRI experiment, mice were anesthetized using urethane (typically a 36% solution, ip, 0.083 ml/20 g animal weight, prepared fresh daily; Aldrich, Milwaukee, WI). To maintain the core temperature, a recirculating heated water blanket was used. Rectal temperatures were continuously monitored throughout each experiment, as previously described [11].

The left eye of each animal was examined using a 1.0 cm diameter coil, which was positioned such that the eye protruded slightly through its center. Note that MEMRI measures the cumulative intraretinal uptake of manganese after the injection (while the animal is awake and freely moving in light or dark conditions).

A single transverse slice, obtained using an adiabatic pulse sequence [12], positioned in the middle of the lens and middle of the optic nerve (i.e., through the center of the eye) was collected using the 4.7 T system. High-resolution images were acquired using an adiabatic spin-echo imaging sequence (repetition time TR 350 s, echo time TE 16.7 ms, number of acquisitions NA 16, matrix size 512x512, slice thickness 620 μm, field of view 12x12 mm^2^, 54 min/image) [12]. Following the MEMRI exam, the animals were euthanized.

### MRI data analysis

**Figure 1:**
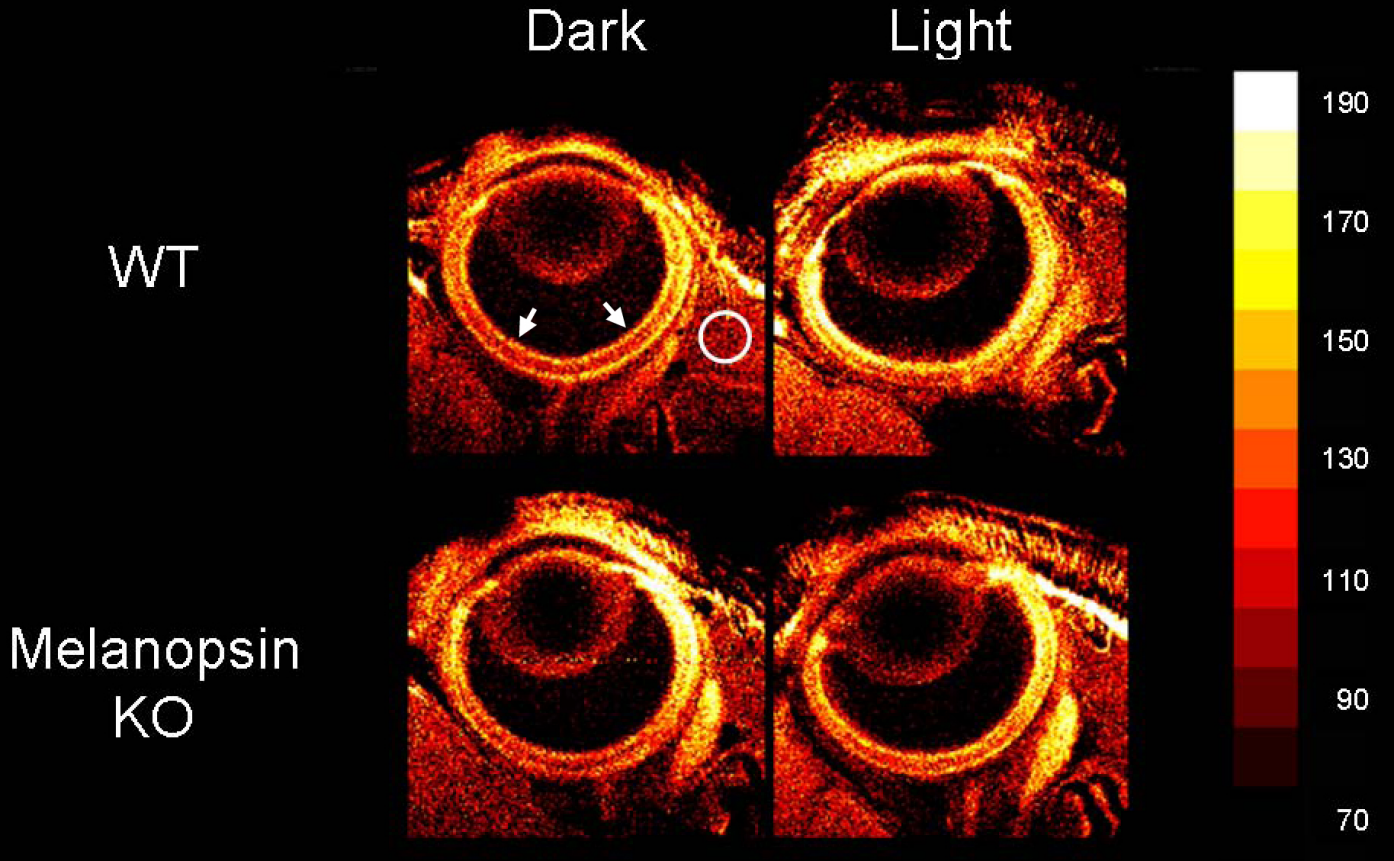
Representative manganese-enhanced MRI (MEMRI) data of P7 wild type (WT; top row) and melanopsin knock out (KO; bottom row) mouse eyes following either dark (left column) or light (right column) adaptation. The white circle indicates the region of extraocular muscle used to normalize retinal signal intensities in this figure and in Figure 2. The same muscle-normalized signal intensity scale (far right) was used for all images and in all analyses. Retinal signal intensity data in Figure 2 were extracted from the central retina (0 to 30% of the retinal extent or the region between the arrows). The width of each image is 4.14 mm.

In each image, intraretinal signal intensities (SIs) were normalized to the SI of a fixed-size region-of-interest drawn in the anterior-most aspect of the adjacent muscle (Figure 1). For this step, which reduces inter-individual variability due, for instance, to differences in surface coil placement, the same section of muscle was used throughout. In a first-pass analysis, retinal ganglion cell/inner plexiform layer SIs were analyzed using the program NIH IMAGE and derived macros. Retinal SI data were extracted 0.4 and 1 mm on either side of the optic nerve (i.e., superior and inferior retina, Figure 1) from the presumptive ganglion cell / inner plexiform layers (i.e., 47–70 μm (i.e., 2–3 pixels) posterior to the clearly defined vitreoretinal division) and were analyzed as previously described [8]. To further characterize SIs across all retinal layers, we first used software written in-house to map the in situ image into a linear representation for each retina, as described previously [6]. To facilitate group comparisons, the distance from the ora serrata to the optic nerve head was measured in each hemiretina and used to bin linearized data by eccentricity into ten equal segments per hemiretina. For each bin, the average profile of SI as a function of retinal depth was calculated and the vitreous-retina (0%) and retina-choroid (100%) borders were found using the “half height” method [13]; the distance between these two borders is the whole retinal thickness. The SI profiles for each bin were then re-sampled according to relative distance between those borders. At the end of processing, retinal data were spatially normalized in both dimensions (% retinal extent -by- % retinal thickness). Data from the central retina (0 to 30% of the panretinal extent) was averaged for statistical analysis of retinal thicknesses and SIs.

### Statistical analysis

In the first-pass analysis, comparisons of MEMRI retinal SIs between groups were performed using a generalized estimating equation (GEE) approach to compare data extracted from the retinal ganglion cell/inner plexiform layers. This approach involves a general linear regression analysis using all the pixels in each subject and accounts for the within-subject correlation between adjacent pixels. A p<0.05 was considered statistically significant for these light-dark comparisons, as well as for retinal thickness comparisons of control and melanopsin-knockout mice, which were conducted using two-tailed *t*-tests.

**Figure 2:**
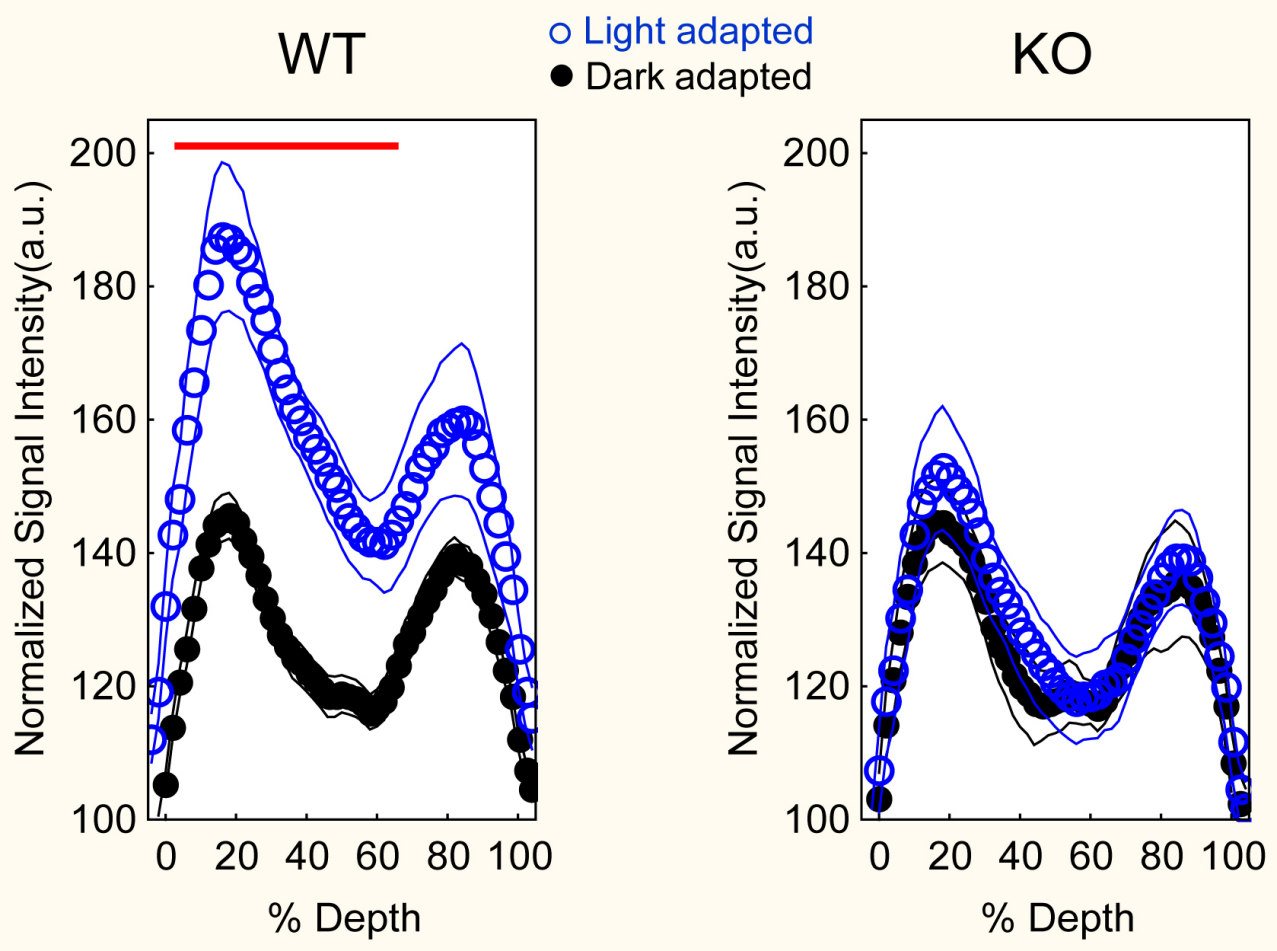
Retinal signal intensity data, normalized to muscle, for light (blue symbols (means) and lines (SEMs)) and dark (black symbols (means) and lines (SEMs)) plotted as a function of distance from the vitreous-retina border (0%) to the retinal-choroid border (100%) for wild type (WT; left graph) and knock out (KO; right graph) mice. The red line indicates the retinal region demonstrating a significant difference (p<0.05) between light- and dark-adapted WT mice.

To analyze group differences in Mn^2+^ uptake across the retina, ANOVAs were performed at each distance from the vitreoretinal division (0% in Figure 2). To limit false positives among these multiple comparisons, only those ANOVA results falling below a false discovery rate threshold of q=0.05 (calculated with c(V)=1; [14]) were considered statistically significant. Where ANOVA results were significant, between-group differences were evaluated using a Tukey’s Honestly Significant Difference test and an adjusted p value of <0.05 was considered significant. Data are presented as mean±SEM, unless otherwise noted.

## Results

Visual inspections of representative images of each group investigated suggested greater manganese accumulated in light-adapted retinas of WT mice compared to dark-adapted WT mice, as well as in light- and dark-adapted KO mice (Figure 1). Note that in this study, no canthotomy was performed on any of the pups, as illustrated by the intact eyelids in Figure 1.

Quantitative analysis supported this impression. Intraretinal uptake of manganese in WT mice increased (p<0.05) with light adaptation (e.g., entire intraretinal mean SI 157±8 a.u. [arbitrary units]) relative to that in the dark (mean 128±3 a.u.) (Figure 2). In KO mice, no significant (p>0.05) difference in uptake was noted between light- (mean 127±6 a.u.) and dark- (mean 131±7 a.u.) adapted mice (Figure 2). Through much of the retina, significantly more Mn^2+^ uptake was found in light-adapted WT animals than in dark-adapted WT mice (0%–64% depth, p<0.05; Figure 2), dark-adapted KO mice (0%–64% depth, p<0.05), and light-adapted KO mice (0%–60%, except at 6% and 10%, p<0.05). There were no differences (p>0.05) between light-adapted KO mice, dark-adapted KO mice, and dark-adapted WT mice.

No differences (p>0.05) in whole retinal thickness were found between the dark-adapted WT (257±5 μm), light-adapted WT (249±10 μm), dark-adapted KO (262±10 μm), and light-adapted KO (244±8 μm) mice.

## Discussion

In this study, intraretinal uptake of manganese was greatest in light-adapted WT P7 mice compared to dark-adapted animals. This light-dark difference in uptake was absent in the melanopsin KO mice. The different control and KO strains used are not considered a confounding factor since a similar light-induced increase in retinal manganese uptake, relative to that in the dark, was also noted in preliminary control P7 Sprague Dawley rat studies (data not shown). The data in Figure 1 and Figure 2 are consistent with melanopsin-mediated effects extending at least partly into the presumptive inner nuclear layer [15]. Together, our data demonstrate for the first time the feasibility of using MEMRI to measure melanopsin-mediated light responses in young mouse retinas in vivo. More work is now needed to analytically evaluate how, for example, the extent of retinal manganese uptake in young rodents is related to circadian rhythm, and how calcium channel activity in other retinal layers is being regulated by melanopsin.

The present study has two potential limitations. First, in-plane spatial resolution of the MEMRI images is such that one pixel (23.4 μm) spans about 11% of the whole retinal thickness. Thus, some partial-volume averaging is expected and is further exacerbated by the 620 μm slice thickness. This partial-volume averaging somewhat confounds attempts to produce a detailed map showing the specific retinal layers that are modulated by light and melanopsin. However, this partial-volume averaging cannot account for the apparent light- and melanopsin-dependent increase in manganese accumulation in the outer retina (at 64% of the whole-retinal thickness) if the influence of light and melanopsin is only confined to the ganglion cell layer. We reason that other retinal layers must be involved either directly via melanopsin activity or indirectly via connections with intrinsically photosensitive ganglion cells. Higher resolution data are now needed to fully interpret these results.

A second limitation of this study involves the temporal resolution of the MEMRI method. In this case, functional information was encoded over 4 h. One consequence of this low time resolution is that MEMRI is not able to detect individual spontaneous and periodic bursts of action potentials in P7 mice [16]. These bursts result in large waves of increased intracellular calcium concentration across the retina and likely contribute to retinal manganese accumulation in all groups in the present study [16]. Nonetheless, data available through the MEMRI method provides a wider perspective from which to interpret the global influence of spontaneous activity by measuring the time-average of hundreds of these waves.

Unlike the present results in young rodents, in adult WT and *rd1/rd1* mice [17], inner retinal manganese uptake is not a function of light adaptation [8,18]. This is not unexpected since, for example, melanopsin expression and melanopsin-positive cell density undergo marked decline in mature retina relative to that in young animals [1].

Furthermore, in adult WT mice, changes in inner retina activity caused by light or dark adaptation would be expected to be relatively equal due to similar representation of ON and OFF cells (bipolar and ganglion cells) [8,18]. An alternative, and possibly complementary, interpretation of the present data are that ipRGCs regulate the frequency of spontaneous retinal waves and that light adaptation causes the waves to increase in frequency [19]; the absence of spontaneous activity and their associated large ion fluxes might also be why no light-dark differences in manganese accumulation are detected in the inner retina of adult mice. More work is needed to understand the importance of these various modulating factors in understanding the MEMRI data. In any event, MEMRI study of melanopsin light-dependant activity in rodents appears limited to early postnatal development.

It is not yet known if manganese ions directly permeate the melanopsin channel. Light activates melanopsin-mediated calcium ion influx, largely via voltage-gated calcium channels [3]. This route likely contributes to neuronal manganese accumulation in the present study: Mn^2+^ is a Ca^2+^ surrogate and readily permeates voltage-gated calcium channels [20]. Future studies using specific calcium channel antagonists will likely be able to further identify the type of calcium channels involved with manganese accumulation [21,22]. Interestingly, one downstream consequence of voltage-gated calcium channel activation is increased c-fos expression, a commonly used metric for evaluating light-induced melanopsin activity [1,23,24]. Consistent with this link, activity measurements using MEMRI and c-fos co-localize in the rodent brain [24]. While the two approaches are complementary, MEMRI, despite its relatively lower spatial resolution, has at least one advantage over c-fos: it can non-invasively evaluate retinal function in the same animal over time.

In summary, first time evidence is presented that demonstrates the unique use of MEMRI as a useful approach for analytically evaluating the activity of melanopsin in the retina.
